# Classical music restored fertility status in rat model of premature ovarian failure

**DOI:** 10.1186/s12906-022-03759-y

**Published:** 2022-11-09

**Authors:** Nahideh Nazdikbin Yamchi, Mohammad Mojtaba Alizadeh Ashrafi, Hamed Abbasi, Farhad Amjadi, Mohammad Hossein Geranmayeh, Reza Shirazi, Amin Tamadon, Reza Rahbarghazi, Mahdi Mahdipour

**Affiliations:** 1 Stem Cell Research Center, Tabriz University of Medical Sciences, Tabriz, Iran; 2 Faculty of Design, Tabriz Islamic Art University, Tabriz, Iran; 3 Faculty of Lyrical Literature, Islamic Azad University, Arak, Iran; 4 Mehr Afarin Ahang, Cultural-Artistic Co, Tehran, Iran; 5 Neurosciences Research Center (NSRC), Tabriz University of Medical Sciences, Tabriz, Iran; 6 Department of Anatomy, School of Medical Sciences, Medicine & Health, UNSW Sydney, Sydney, Australia; 7 Percia Vista R&D Co., Shiraz, Iran; 8 Department of Applied Cell Sciences, Faculty of Advanced Medical Sciences, Tabriz University of Medical Sciences, Tabriz, Iran; 9 Department of Reproductive Biology, Faculty of Advanced Medical Sciences, Tabriz University of Medical Sciences, Tabriz, Iran

**Keywords:** Premature ovarian failure, Music therapy, Sex-related hormones, Brain activity, Ovarian rejuvenation

## Abstract

**Background:**

The restorative effect of classical music was assessed on the cyclophosphamide-induced animal model of premature ovarian failure (POF).

**Methods:**

Mozart’s piano classical music (K.448) was used for up to 4 and 8 weeks. Rats were exposed to music 6 h every day using a stereo system with a volume of 65–70 dB. Sera and ovarian tissue samples were collected for the evaluation of FSH, LH, and E_2_ and histopathological examination. At the same time points, samples were taken from the hypothalamus and hippocampus to monitor the expression of *Ntrk2*, *Crh*, and *Pomc* using real-time PCR. Mating trial was performed to evaluate the fertility status of POF rats.

**Results:**

Histopathological examination revealed a significant increase (*p* < 0.05) in the numbers of morphologically normal follicles at all the developmental stages in POF rats after music therapy compared to the POF group (*p* < 0.05). Music therapy decreased FSH and LH levels to near-to-normal levels conidied with elevation of E_2_ (*p* < 0.05). *Ntrk2*, *Crh*, and *Pomc* expressions were down-regulated in POF rats. Music therapy increasaed the expression of *Ntrk2* in the hypothalamus of POF rats (*p* < 0.05*)*. In contrast, *Crh* and *Pomc* failed to reach the detection limit before intervention and four weeks after the intervention however, these genes were expressed eight weeks after music therapy. Fertility status was increased (*p* < 0.05) in terms of litter size in POF rats after being exposed to music compared to the non-treated POF control group (*p* < 0.05).

**Conclusion:**

Results showed that music can exert therapeutic effects on POF rats via the alteration of sex-related hormones.

**Supplementary Information:**

The online version contains supplementary material available at 10.1186/s12906-022-03759-y.

## Introduction

Premature ovarian failure (POF) is defined as an ovarian dysfunction, leading to the alteration of the development of ovarian follicles [1, 2]. Statistics have revealed that POF affects about 1% of women under 40 years old and about 0.1% of women under the age of 30 [3]. Both environmental and genetic factors are noted to cause POF, whereas chromosomal defects (fragile X syndrome), toxins (including chemotherapy and radiotherapy), autoimmune diseases, infections, and thyroid malfunction are reported to alleviate this disorder [3, 4]. In the POF patients, follicle-stimulating hormone (FSH) levels reach above 40 IU/mL while the content of anti-Mullerian hormone (AMH) declines below 1 ng/mL [5]. Different clinical symptoms such as amenorrhea, hypoestrogenism (estrogen reduction), and hyper-gonadotropism (increased levels of gonadotropins) can be manifested in POF women [6]. Until now, various pre-clinical strategies have been developed for the treatments of POF including hormone replacement therapy (HRT), gonadotropin-releasing hormone (GnRH) application, and modalities that are associated with the application of whole-cell, or cell-based products [7–14].

Despite the recent progress in the alleviation of POF, there is a great deal of emphasis on the use of complementary therapies. Among them, music therapy is at the center of attention. Music therapy has a long history and dates back to the writings of Plato, Pythagoras, and Aristotle, who were all aware of the power of prevention and treatment of music [15]. It has been indicated that this approach can lead to relaxation, accelerate the healing process of diseases, and improve mental function [16–18]. Music exerts its effects through the coordination of different rhythms of the body and regulates physiological responses in different ways [19]. Music can stimulate the pituitary gland to release several hormones into the nervous system and bloodstream [20]. Among the musical genres, the physiological and behavioral effects of classical music were studied in rats [21]. Besides, studies have been conducted to investigate the effect of music on anxiety and biological factors using workpieces of musicians such as Bach, Beethoven, Mozart, etc. [22, 23]. Classical music therapy has been practiced successfully on mice animal models for stress [24–26], diabetes mellitus [27], breast cancer [28], bone cancer [29], Alzheimer’s disease [20], autism [30], and schizophrenia [31].

Various signaling molecules are associated with POF including *Ntrk2* in which loss of the *Ntrk2*/*Kiss1r* pathway in oocytes has been shown to cause POF conditions [32]. Previous studies have further shown this gene besides its unique influence on the development of the nervous system, is involved in controlling ovarian function. Thus, the ovaries of mice lacking *Ntrk2* receptors show fewer primary follicles and causing deficiency in early follicular growth [33]. Also, the components of the corticotrophin-releasing factor (CRH) family, as a stress hormone receptor system, helps both initiate stress responses and restore systems to homeostasis after the removal of the stressor [34]. CRH can regulate steroidogenesis which is involved in follicular maturation, ovulation, and luteolysis [35]. Also, Proopiomelanocortin (POMC), is a precursor protein detected in the female reproductive system, from which peptides are synthesized in the ovary, and has been confirmed to play a significant role in ovarian function [36, 37].

Considering the positive effects of music therapy in various pathological conditions, in this study, we investigated the restorative effects of non-invasive music therapy on an experimentally induced rat model for POF. To this end, we explored the follicular counts, hormonal alterations as well as the expression of transcripts in the favor of regeneration of ovarian tissue and fertility preservation.

## Materials and methods

### Animal ethics

Here, 23 female Wistar rats (7–8 weeks old), weighing between 150 and 180 g, were purchased from Med Zist Company-Tehran. Rats were housed in the normal environment with a temperature of 22 ± 2 °C and 12 h of light/dark cycle and free access to standard pellet and water. Before the experiments, rats were kept untreated for 1 week for environmental adaptation. All experimental protocols were confirmed by the local ethics committee of Tabriz University of Medical Sciences (IR.TBZMED.VCR.REC.1398.361).

### Production of rat model of premature ovarian failure

**Fig. 1:**
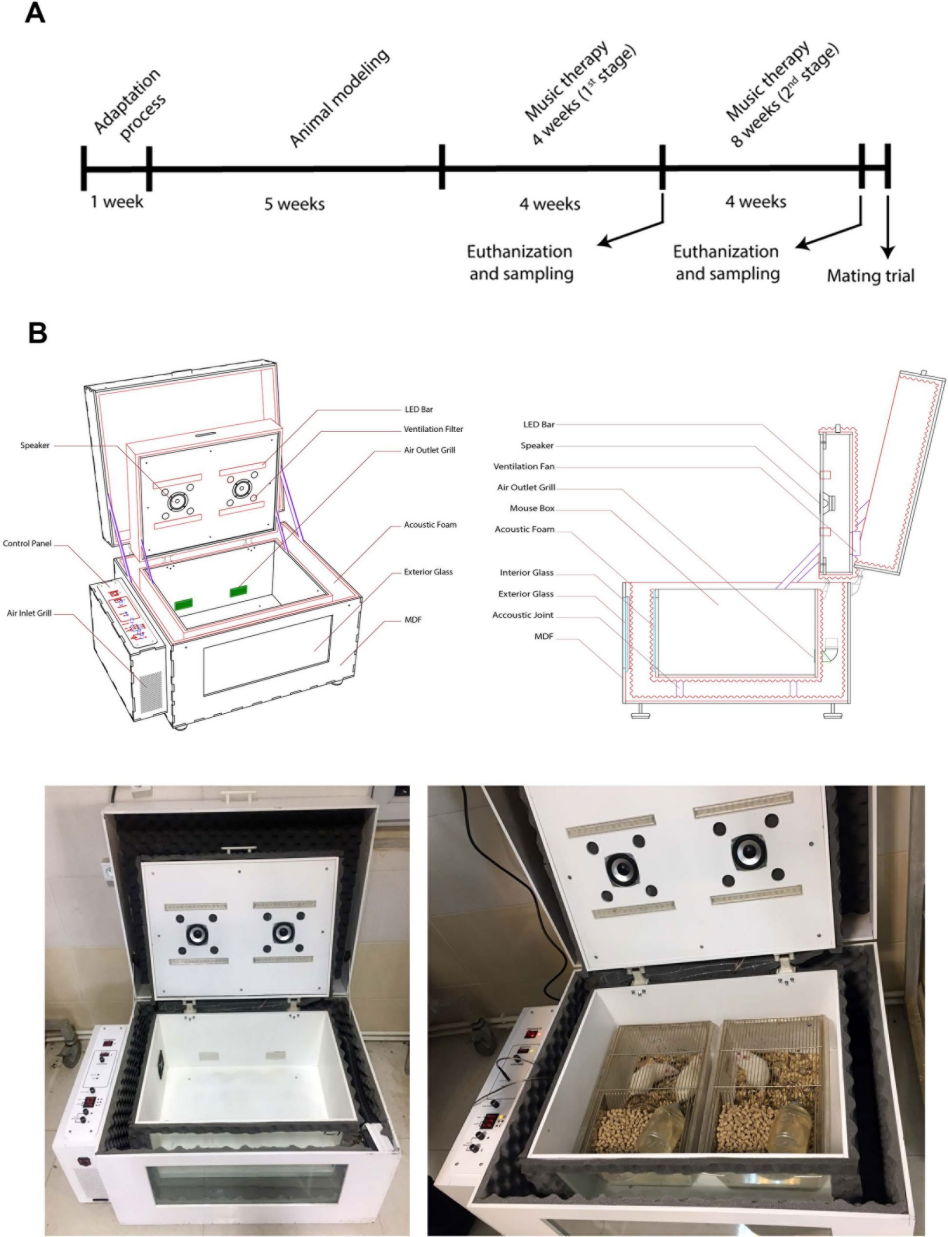
Timeline of the experimental procedure (**A**), and the design and development of acoustic music box (**B**)

To induce the rat model of POF, 20 rats were subjected to the intraperitoneally (IP) administration of cyclophosphamide (CTX; Cat no: RHRI404, Supelco) at a dose of 200 mg/kg on day 1 and 8 mg/kg on days 2 to day 14. CTX is an active substance and can destroy follicles by the mechanism of apoptosis and tissue necrosis [38]. According to previous protocols, 21 days after the last injections rats can exhibit POF features [39]. To confirm POF status, 3 rats were randomly selected from both POF and the control groups and euthanized using an overdose of Ketamine and Xylazine. The left 18 rats were arbitrarily allocated into Control, POF, and POF plus music therapy. The rats in the experimental group were kept in the music box for 4 and 8 weeks (Fig. 1A).

### Acoustic music box

To make an enclosed environment with an adequate rate of ventilation, illumination, and temperature, we designed an insulated acoustic box equipped with a control panel to adjust light, ventilation, and sound systems ﻿similar to room conditions (Fig. 1B). The sound speakers were connected from outside to an mp3 stereo playback system. Before playing the music, the volume was measured with a decibel meter. Finally, Mozart’s Piano Classical Music (K 448) was played for 6 h every day from 4 to 10 pm with a volume of 65–70 decibel (dB) up to 4 and 8 weeks. The developed box could hold two cages.

### Tissue and blood sampling

For euthanization, an overdose of Ketamine and Xylazine was administrated IP. Blood was taken directly from the heart to investigate the serum levels of hormones. For histopathological examination, tissues were collected, rinsed in phosphate-buffered saline (PBS) solution, and fixed in 10% formalin (Merck). For real-time PCR analysis, brain tissues (hypothalamus and hippocampus) were individually sampled in cryovials and stored at -80 until the analyses.

### Histopathological evaluation

Formalin-fixed ovarian specimens were embedded in paraffin after dehydration in alcohol series and cut to a thickness of 5 μm using a microtome instrument (Leica). Hematoxylin and eosin (H&E) staining was performed to study the numbers and the quality of follicles at different developmental stages and corpus luteum (CL) [40]. Masson’s trichrome staining was also executed to evaluate collagen fiber deposition as a sign of tissue fibrosis [41]. After staining, follicular populations and the presence of collagen fibers were evaluated under an Olympus BX-51 light microscope and recorded using a digital camera.

### Measurement of serum levels of FSH, LH, and E_2_

To measure serum levels of FSH, LH, and E_2_, blood samples were clotted in glass tubes and serum was collected after centrifugation for 20 min at 400xg and stored at -80 °C. Enzyme-linked immunosorbent assay (ELISA) method was performed using a commercial kit for measuring the levels of FSH (0334 − 96, Monobind), LH (0234 − 96, Monobind) and E2 (4925-300 A, Monobind(.

### Real-time PCR assay

To evaluate the expression of *Ntrk2*, *Crh*, and *Pomc*, hypothalamus and hippocampus samples were subjected to RNA isolation according to the protocol (Traysol: 0000124, MaxZol). Then, the RNA was reverse-transcribed to cDNA (cDNA synthesis kit; YT4500, Yekta Tajhiz Azma). Specific primer pairs were designed using online software (www.ncbi.nlm.nih.gov/tools/primer-blast/) by considering different variables for each gene (Table 1). Subsequently, a quantitative real-time polymerase chain reaction (qRT-PCR) was performed using cDNA and SYBR Green 2 × (5,000,850, Ampliqon) with the “Roche Light Cycler 96” system. The annealing temperature was detected using a gradient PCR. The PCR reaction program was performed in 45 cycles with denaturation, annealing, and extension (95, 60, and 72 °C respectively all last for 15 s). Finally, the specificity of each reaction was evaluated by analyzing the melting and propagation curves.

**Table 1:** Primers sequences designed for Real-time PCR

Gene/ NCBI accession number	Sequence (5’→3’)		Annealing Temperature (˚C)
***Ntrk2*** **NM_001163168.2**	F	ACCTGCGGCACATCAATTTC	60
	R	ACAAATCCTGAGTGTCGGGG	60
***Crh*** **NM_031019.2**	F	CTGATCCGCATGGGTGAAGA	60
	R	GGAAAAAGTTAGCCGCAGCC	60
***Pomc*** **NM_139326.3**	F	ATAGACGTGTGGAGCTGGTG	60
	R	CGGAAGTGACCCATGACGTA	60

### Assessment of fertility status

Finally, the remaining three rats from both POF + music and POF control groups were mated with fertility-proven male rats in a ratio of 2:1 to assess their reproductive status. After examining the vaginal plug, the rats were placed individually in separate cages for 3 weeks. Finally, the litter size/rat was registered.

### Data analysis

All the results presented in mean ± SEM were examined by Graph Pad Prism 8 software. To evaluate the statistical significance between groups, data were analyzed with one-way ANOVA with a post-hoc test (Fisher’s least significant difference, LSD). The student’s t-test was incorporated to analyze the significant differences between the two groups. P-values less than 0.05 were considered significant.

## Result

### Rat model of POF was successfully established

**Fig. 2:**
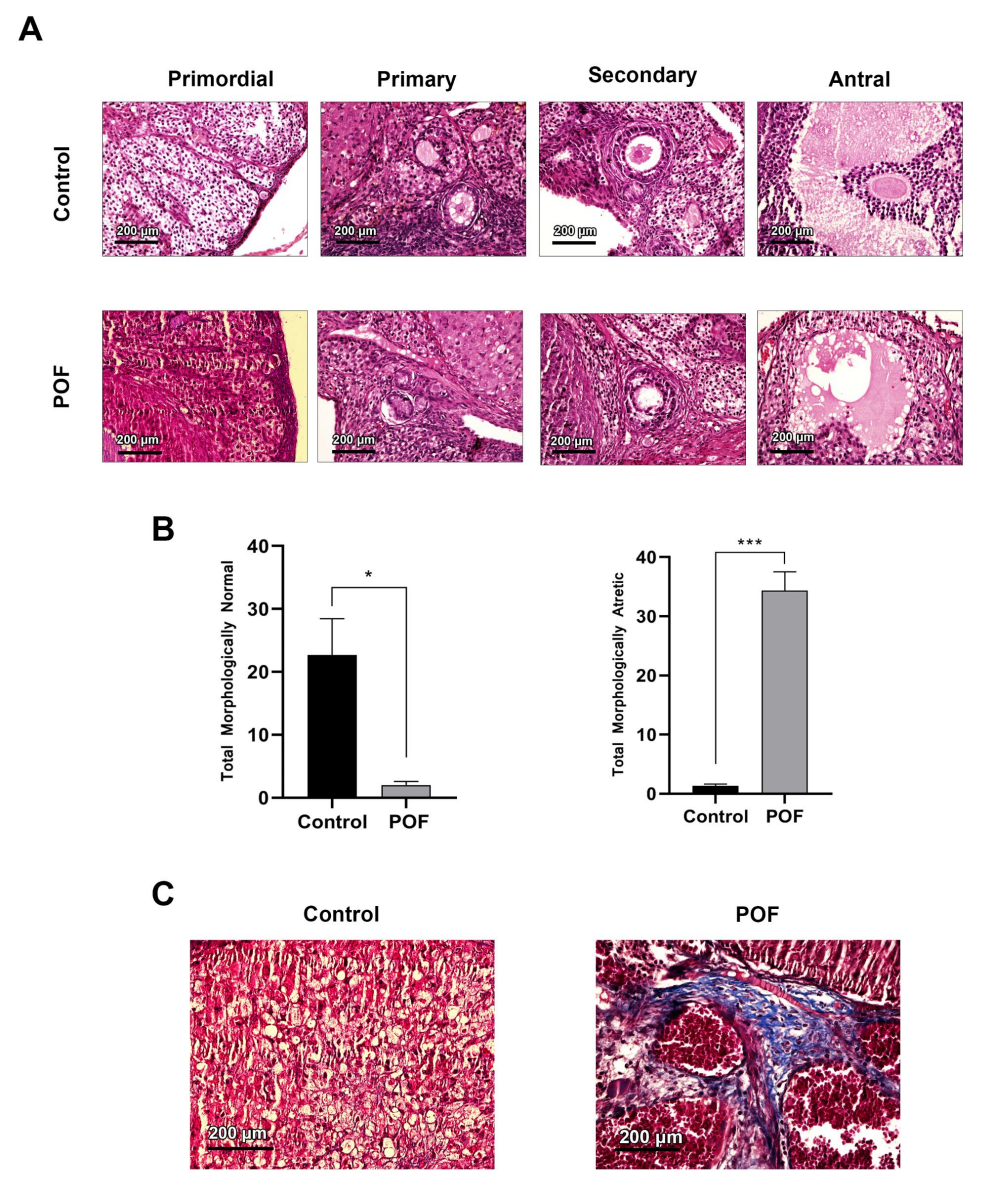
Morphological assessment of follicles before the music intervention, H&E staining of ovarian tissue in control and POF groups before intervention (**A**), total number of follicles (**B**), and Masson trichrome staining after POF production (**C**). *p < 0.05; and ***p < 0.001 (n = 3)

Following CTX injection, H&E staining was performed. The general follicular atresia was noted in the POF rats compared to the control group. The number of morphologically healthy follicles at all developmental stages was significantly declined in the POF rats (*p* < 0.05). Our findings illustrated that the CTX has successfully induced morphological conditions similar to the POF features (Fig. 2A, B). Masson trichrome staining also revealed general collagen fiber deposition within the ovarian tissue of the POF rats (Fig. 2C).

### Music therapy improved ovarian function in POF rats

**Fig. 3:**
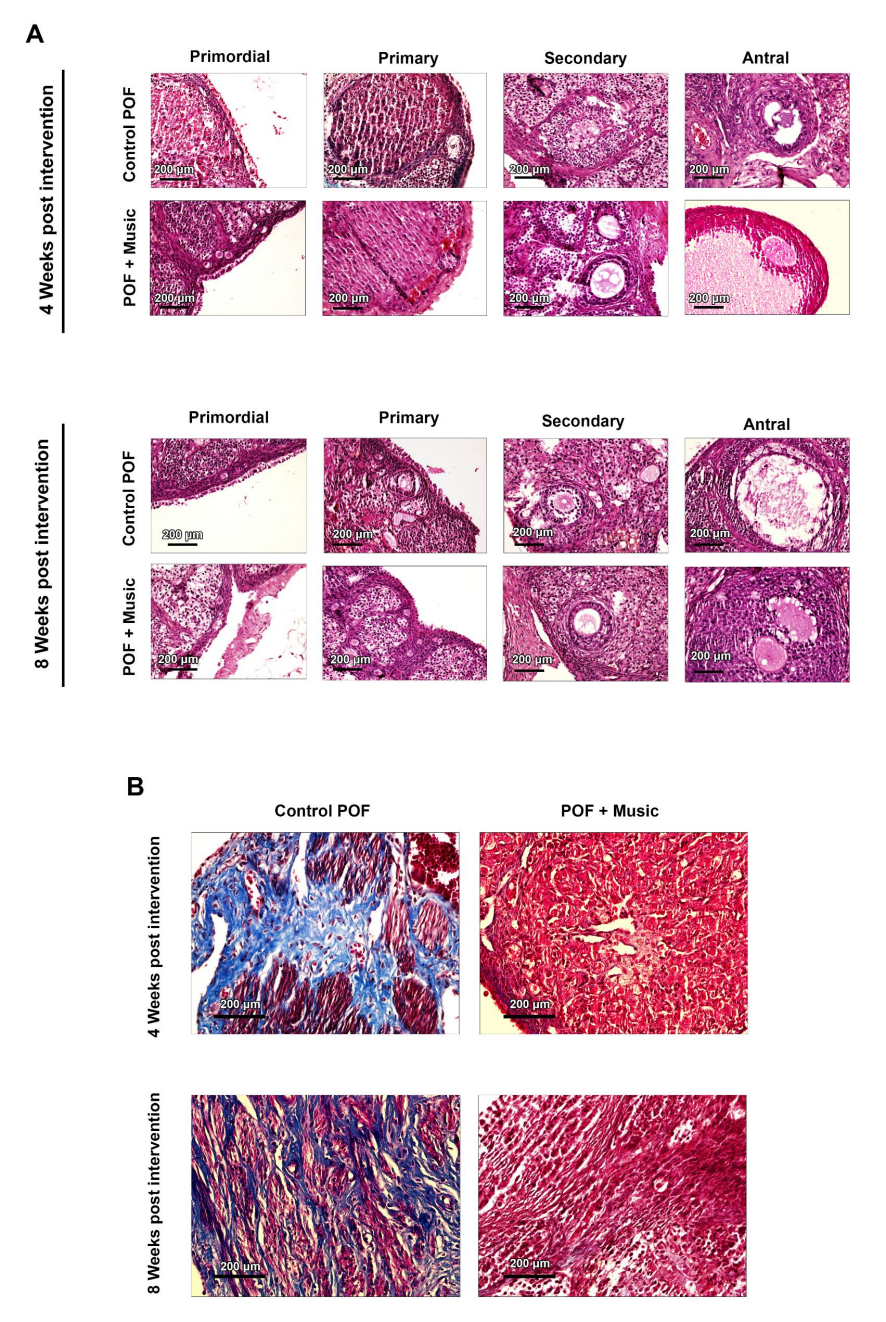
Morphological assessment of follicles 4 and 8 weeks post-intervention, H&E staining (**A**), and Masson trichrome staining (**B**) of ovarian tissue in control and POF groups after intervention

**Fig. 4:**
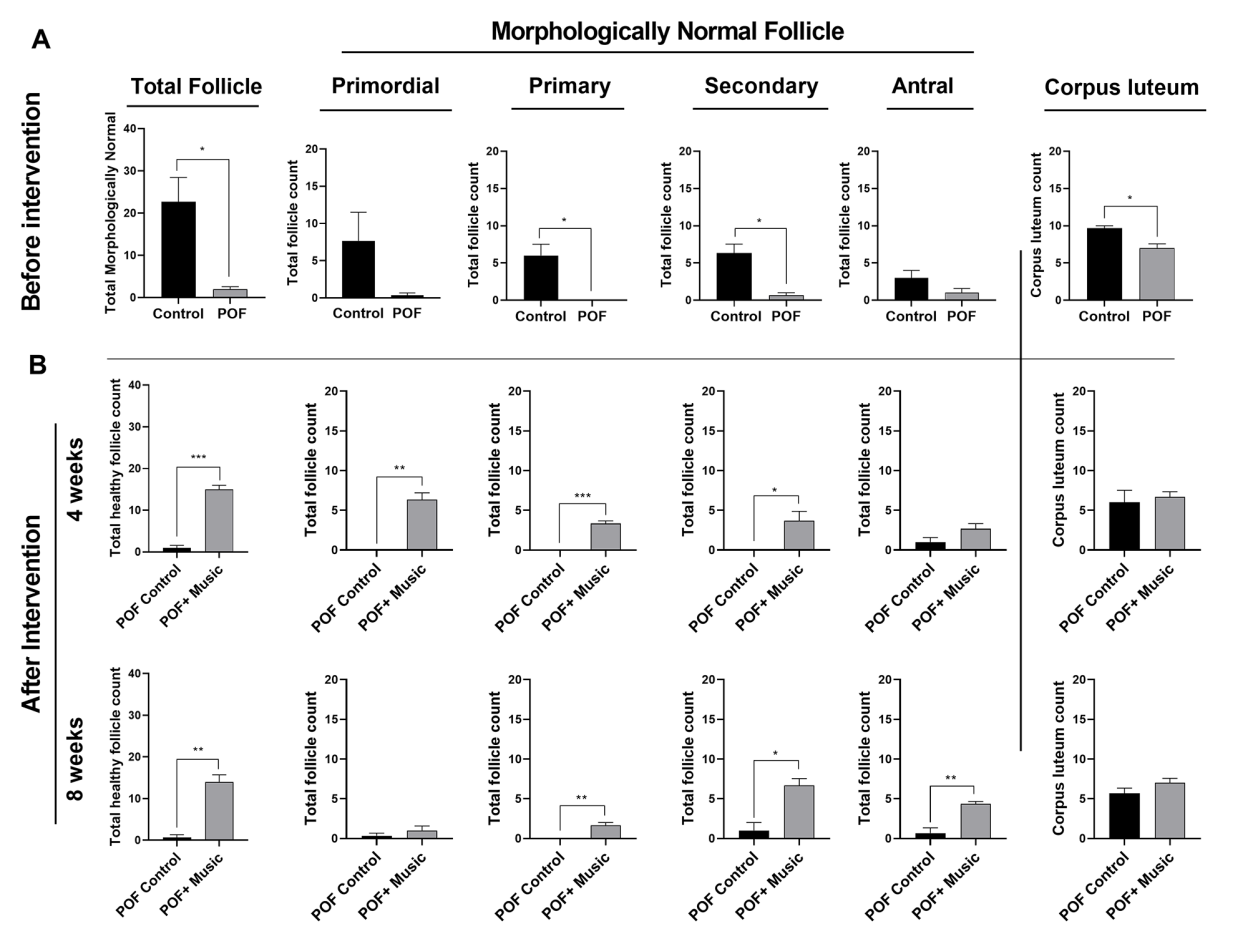
Accurate counting of different types of morphologically normal follicles before (**A**), after receiving music (**B**), and corpus lutetium (**C**). One-way analysis of variance (ANOVA) and least significant difference (LSD) post-hoc analysis. *p < 0.05; and **p < 0.01 ***p < 0.001

We also examined the follicle population of morphologically healthy and atretic follicles after the intervention. Our findings showed that the total number of morphologically healthy follicles was increased significantly in the music group four and eight weeks after exposure to classical music compared to the control POF rats (*p* < 0.01and *p* < 0.001 respectively; Fig. 3B). Similar to what we observed before starting the intervention (Fig. 3A). In contrast, the total number of atretic follicles significantly declined after intervention (*p* < 0.01; Fig. 4A). Further looking at follicular development stages of primordial, primary, secondary, and antral, a general significant improvement in the numbers of morphologically healthy follicles was noted. According to our data, the number of atretic follicles was increased following the induction of POF in rat ovarian tissue. This pattern was reversed 4 and 8 weeks post-music therapy (Supplementary Fig. 1). Our results showed that POF rats had a relatively lower number of CL compared to the control healthy rats (*p* < 0.05; Fig. 3 C). After exposure to music, the number of CL was increased. However, the differences were not statistically significant (*p* > 0.05; Fig. 3 C). In terms of fibrotic changes, our results showed the reduction in collagen fiber deposition in ovarian tissue of POF rats 8 weeks after being exposed to music (Fig. 4B).

### Music therapy altered serum levels of FSH, LH, and E_2_

**Fig. 5:**
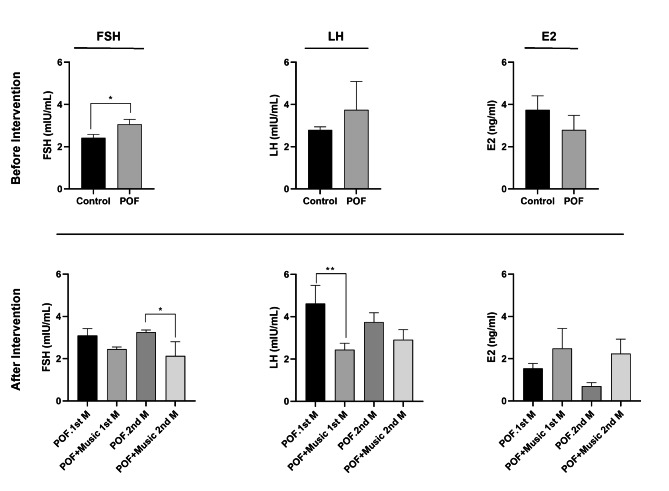
Serum levels of follicle-stimulating hormone (FSH), luteinizing hormone (LH) and estradiol (E_2_), before also 4 and 8 weeks after receiving music. One-way analysis was expressed. Analysis of variance (ANOVA) and least significant difference (LSD) post hoc analysis. *p < 0.05 (n = 3)

We examined the serum levels of FSH, LH, and E_2_ hormones in POF rats before and after music intervention. In POF rats, FSH level was elevated significantly in response to the CTX administration (*p* < 0.05). Along with these changes, LH and E_2_ levels were increased and decreased respectively, however, the differences were not statistically significant (*p* > 0.05). In POF rats exposed to music therapy, serum levels of FSH were statistically significant differences at week 8 (*p* < 0.05). A significant decrease pattern was also noted in terms of LH levels (*p* < 0.01) at week 4 post music intervention. Despite the increase of E_2_ in music-treated POF rats at both time points, the changes were not statistically significant (Fig. 5).

### Music therapy altered the expression of genes involved ovary function

**Fig. 6:**
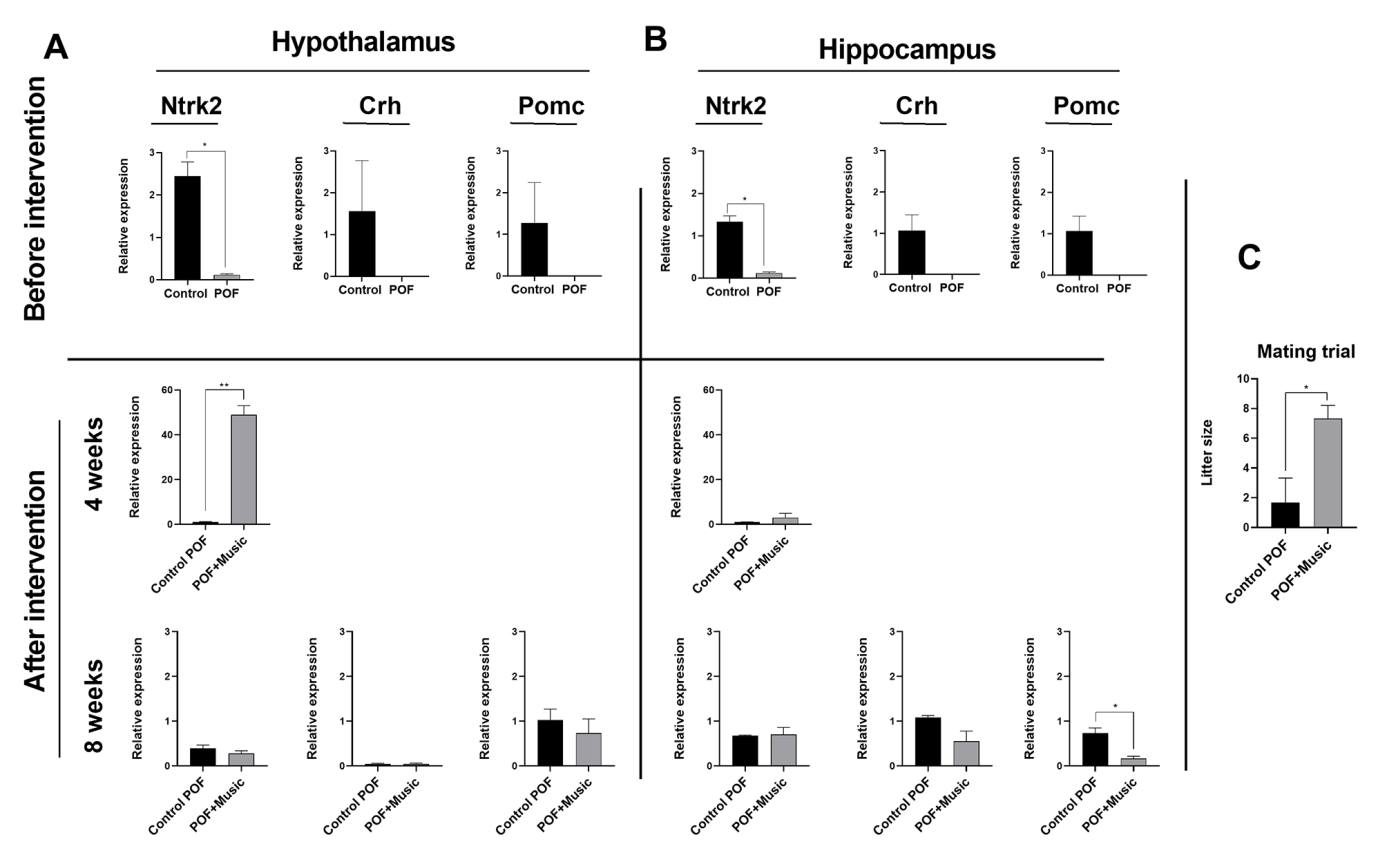
Relative expression of *Ntrk2*, *Crh*, and *Pomc* genes in the hypothalamus (**A**), and hippocampus (**B**) before also 4 and 8 weeks after receiving music. One-way analysis of variance (ANOVA) and least significant difference (LSD) post hoc analysis. *p < 0.05; and **p < 0.01 (n = 3). Litter size at 8 weeks post-intervention (**C**)

Real-time PCR results showed that *Ntrk2*, *Crh*, and *Pomc* genes were down-regulated in hypothalamus and hippocampus tissues following the induction of POF. Notably, the differences were only statistically significant for *Ntrk2* (*p* < 0.05; Fig. 6). In the hypothalamus, *Ntrk2* expression was interestingly up-regulated 4 weeks after music therapy (*p* < 0.01). In contrast, both *Crh* and *Pomc* genes did not reach the detection limit at this time point. Nevertheless, non-significant differences were noted regarding the expression of subjected genes in the hypothalamus 8 weeks after music therapy (Fig. 6 A). In hippocampus tissue, only the expression of the *Ntrk2* gene was detected 4 weeks after therapy without significant changes compared to the POF rats (*p* > 0.05). Eight weeks after music therapy, *Crh* and *Pomc* expression were down-regulated with only significant changes for *Pomc* (*p* < 0.05) (Fig. 6B).

### Music therapy improved the fertility status of POF rats

To evaluate the fertility status in rats, three remaining rats from the music group and three rats from the POF control group were mated to examine the number of offspring eight weeks after music therapy. In the music group, rats gave birth to 7, 6 and 9 healthy babies, however, only one rat gave birth to 5 babies which were in total statistically significant when the two groups were compared (*p* < 0.05; Fig. 6 C).

## Discussion

POF is one of the main causes of infertility in different countries with great clinical and economical concerns [42]. To date, routine therapeutics have been not effective enough to restore the function of ovarian tissue [43]. Therefore, various strategies have been practiced to regenerate ovarian tissue including cell and cell-product-based approaches mostly in animal setups [5]. The creation of chemotherapy-induced POF models has received a great deal of attention in recent years. Chemical compounds like Busulfan, Cisplatin, and CTX are shown to cause follicular atresia and depletion in ovarian tissue, mimicking POF-like conditions [44–46]. Here, we successfully induced a rat model for POF using CTX [47]. Biochemical analysis showed that the levels of FSH and LH hormones were significantly increased in the POF conditions. In contrast, the induction of ovarian insufficiency can lead to the reduction of E_2_ [48, 49]. According to changes in serum levels of sex-related hormones, effective treatment should focus on the regulation of these hormones. Based on our data, music therapy can reduce increased levels of FSH and LH in POF rats 8 weeks after treatment with music [50]. Statistically significant differences were notified between FSH, LH, and E_2_ levels in the music-treated POF rats compared to the non-treated POF group. These data showed that music can alter serum levels of sex-related hormones in the POF rats.

Folliculogenesis is an important part of ovarian function and provides oocytes for reproduction [14]. In the POF conditions, the population of healthy follicles declines due to general atresia, leading to massive fibrosis. In the present study, healthy primordial, primary, secondary, and antral follicles were increased in POF rats four and eight weeks after being exposed to music. We also monitored the expression of *Ntrk2*, *Crh*, and *Pomc* in both the hypothalamus and hippocampus tissues. We noted that the POF condition can reduce the expression of these genes in both target tissues whereas even in some cases the expression level did not fall within the detection limit. In line with our findings, various studies have shown that *Ntrk*1, 2 are putative players in controlling ovarian function in addition to developing the nervous system [51]. Likewise, other researchers have reported that *TrkA* and *B* receptors encoded by *Ntrk*1, 2 facilitate follicle accumulation and early follicular growth in rat ovaries whereas ovaries of mice lacking the *Ntrk* gene had fewer primary and secondary follicles [33, 52]. Dorfman et al. stated that deletion of the *Ntrk2* gene in mice oocytes resulted in POF conditions [53]. These findings show that the hypothalamic-pituitary-adrenocortical axis is altered shortly when animals failed to proceed with their normal oogenesis. We found that the expression of *Ntrk2* was up-regulated 4 weeks after music therapy while the expression of *Crh* and *Pomc* were not quantifiable. After 8 weeks, however, the expression reached to close to the control POF group except *Pomc* gene in which significant reduction was noted (*p* < 0.05). Components of the corticotrophin-releasing factor (CRH) family, a stress hormone receptor system, help both initiate stress responses and restore systems to homeostasis after the removal of stressors [34]. This gene has also been identified in the reproductive system (ovary, endometrium, placenta, and testis). In the human ovary, receptors are detected in stromal cells and follicular fluid. CRH regulates the ovary in steroidogenesis and is involved in follicular maturation, ovulation, and luteolysis [35]. In a study by Calogero et al., the *Crh* gene was shown to be able to suppress estrogen production in mouse and human granulosa cells in vitro [54]. The results of our investigation also showed that the *Crh* gene is not expressed in the hypothalamus and hippocampus after the production of the POF model and also four weeks after receiving music, however not significant, expressed eight weeks after music therapy, probably due to the short timing exposure to the music. The expression of *Pomc* transcript expression has been confirmed in the ovary by various studies [37, 55]. In a study conducted by Galinelli and co-workers, the expression of *Pomc* was revealed in the ovaries of women of fertile age to be higher than women in post-menopausal states [37]. Chen et al. noted that the expression of the *Pomc* gene is regulated by gonadotropins in the ovaries, and experiments on rats showed that *Pomc*-derived peptides were more abundant during pregnancy than in immature rats [36]. In this experiment, *Pomc* expression was not quantifiable shortly after induction of POF model similar to *Crh* results. Eight weeks after music therapy, *Pomc* transcript was detected in both target samples in which significant downregulation was registered the hypothalamus tissue of music-treated POF group compared to the control POF rats (*p* < 0.05). This could be probably a sign of tissue rejuvenation as a result of therapy. Finally, the fertility of music-treated mice was assessed after eight weeks. According to the results of previous studies, we also showed that the number of offspring in POF rats exposed to music was more related to the non-treated POF group [9, 12, 56].

## Conclusion

To the best of our knowledge, there is no enough data associated with the therapeutic effects of classical music on the restoration of POF consequences either in animal models or human counterparts. Our findings highlighted the positive effects of music on POF rats done via the improvement of ovarian function in terms of healthy follicles and hormonal activity. Music therapy can facilitate the restoration of fertility and diminish the possibility of tissue fibrosis via the changes in levels of sex-related hormones. It can be proposed that music therapy, as a non-invasive and complementary modality, can be considered as an alternative and/or combined approach for various fertility-related complications notably POF patients.

## Electronic supplementary material

Below is the link to the electronic supplementary material.

Supplementary Material 1

## Data Availability

The datasets used and/or analyzed during the current study are available from the corresponding author on reasonable request.
