# Supplementary figures and images for: Classical music restored fertility status in rat model of premature ovarian failure
Source: BMC Complement Med Ther. 2022 Nov 9;22:290. doi: 10.1186/s12906-022-03759-y (PMC9647984; doi:10.1186/s12906-022-03759-y)

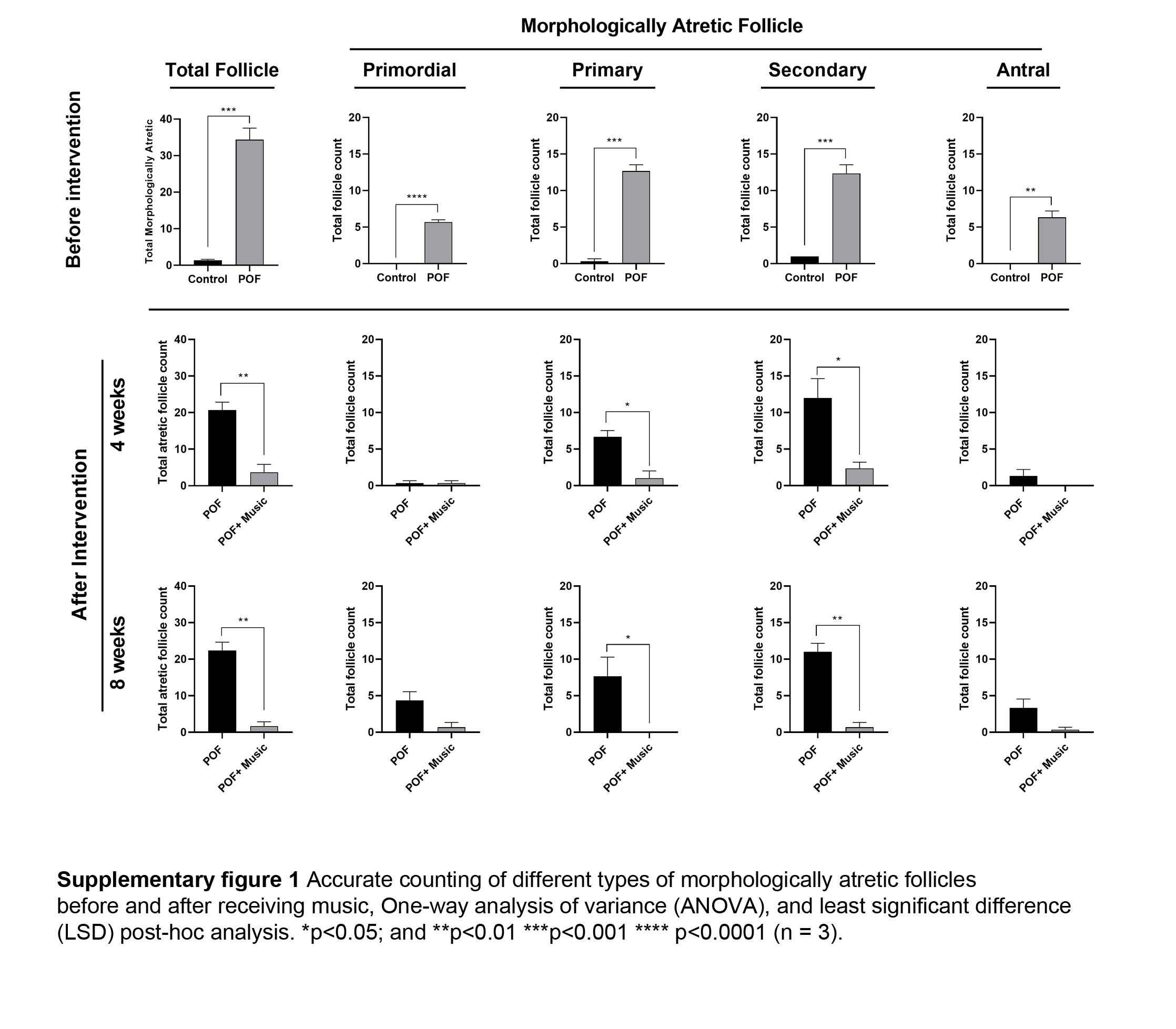

Supplement: Supplementary file 1 — Supplementary Material 1 [file 12906_2022_3759_MOESM1_ESM.jpg]
